# Effects of Ionophores on Ruminal Function of Beef Cattle

**DOI:** 10.3390/ani11102871

**Published:** 2021-09-30

**Authors:** Rodrigo da Silva Marques, Reinaldo Fernandes Cooke

**Affiliations:** 1 Department of Animal and Range Sciences, Montana State University, Bozeman, MT 59717, USA; rodrigo.marques@montana.edu; 2 Department of Animal Science, Texas A&M University, College Station, TX 77843, USA

**Keywords:** cattle, feed efficiency, ionophores, performance, rumen fermentation

## Abstract

**Simple Summary:**

Ionophores are an important nutritional tool used to manipulate ruminal fermentation dynamics and improve the efficiency and performance of beef and dairy animals. Ionophores are carboxylic polyether antibiotics naturally produced by an occurring strain of *Streptomyces spp*. Ionophores modulate the ruminal environment by targeting and altering the metabolism of Gram-positive bacteria, resulting in an increased concentration of ruminal propionate and a reduced acetate concentration. Another pronounced effect of ionophores is the mitigation of ruminal proteolysis and the consequent reduction in ammonia synthesis. The purpose of this review is to highlight the impacts of ionophores on ruminal fermentation, leading to an improvement in the efficiency and performance of beef cattle.

**Abstract:**

Ionophores have been widely used in the beef and dairy industry for decades to improve feed efficiency and performance by altering ruminal fermentation dynamics, increasing the level of propionate. Ionophores can also reduce ruminal proteolysis and ammonia synthesis, thus increasing the influx of protein into the small intestine in cattle, leading to improvements in performance and efficiency responses. Ionophores indirectly impact ruminal methanogenesis by decreasing the substrate used to produce methane. Despite the consistent benefits of using ionophores in cattle nutrition, their utilization is under public scrutiny due to concerns related to microbial adaptation. However, there is inconsistent evidence supporting these concerns, whereas ionophores are still an important dietary tool to enhance productivity and profitability in beef production systems.

## 1. Introduction

Feed additives are an important dietary tool to enhance efficiency and profitability in grazing and feedlot cattle systems [1,2]. Among feed additives, ionophores are the most studied and used in cattle diets, mainly for altering the ruminal microbiome [3,4], optimizing fermentation routes and reducing the rates of digestive disorders [2,5,6]. Changes in the ruminal environment and fermentation dynamics by using ionophores also improve dietary energy and protein utilization [3,7,8]. Ionophores have additional benefits in reducing the formation of foam in the rumen (bloat) and the accumulation of short-chain fatty acids (SCFA), including lactic acid (acidosis), due to the inclusion of rapidly fermentable carbohydrates in the diet [2,5,6,9]. Therefore, ionophores have been used to improve performance, ruminal fermentation parameters, and health of beef cattle. 

Ionophores are carboxylic polyether antibiotics naturally produced by an occurring strain of *Streptomyces* spp and provided to beef cattle orally [9,10]. Ionophores modulate the ruminal environment by targeting and altering the bacterial metabolism of Gram-positive bacteria, such as cellulolytic, proteolytic, methanogenic, and lactate-producing species [11,12,13]. Several ionophores (lasalocid, monensin, salinomycin, laidlomycin, and narasin) are available commercially with a similar mechanism of action in the rumen, whereas animal performance might vary depending on dosage, animal, and diet [1,2,5,9,10,14]. Dietary ionophores can provide the ruminal dynamics with a more efficient fermentation route by altering the ruminal microbiome environment and reducing the substrate for methanogenic bacteria [1,2,4,14,15]. Another noticeable effect of dietary ionophores is mitigating ruminal proteolysis and subsequent reduction in ammonia synthesis [14,16,17]. Hence, the purpose of this review is to provide an overview of the impacts of ionophores on ruminal fermentation of beef cattle.

## 2. Ionophores Mode of Action

Ionophores are carboxylic polyether antibiotics naturally produced by *Streptomyces* spp. bacteria with a very similar mechanism of action in the rumen environment. Russell and Strobel [7] reviewed the ionophores’ mechanism in the rumen, and provided details on the general properties of ionophores and their mode of action. Ionophores are highly lipophilic molecules [18], and their capacity to adhere to bacteria and protozoa membrane determines the vulnerability of the organisms in the gastrointestinal tract. Adherence is, at least in part, determined by the cell wall structure of the bacteria [3,4]. Gram-positive bacteria are absent of protective membrane and, consequently, are more sensitive to ionophores. In turn, Gram-negative bacteria appear to be insensitive to ionophores due to their outer protective membrane, despite such sensitivity mechanisms being poorly understood [19].

Ionophores can interact with metal ions, thereby serving as a carrier for these ions to be transported across the lipid membrane [20]. Most bacteria in the rumen preserve a more alkaline environment by maintaining a high intracellular potassium and a low intracellular sodium concentration [19]. However, the ruminal environment contains high sodium and low potassium, and slightly acidic pH due to SCFA concentrations [7]. Hence, rumen bacteria rely on the ion gradient balance between sodium and potassium to maintain a healthy intracellular environment. Ionophores are metal/proton antiporters that can exchange H+ for either sodium or potassium [7,18]. Once added into the diet, ionophores will insert into the lipid membrane of rumen bacteria, disrupt the intracellular and extracellular ion balance by decreasing intracellular potassium and pH and increasing intracellular sodium [19]. The rumen bacteria react to this intracellular acidification by activating sodium/potassium and hydrogen ATPase systems, which pump these protons out of the cell [21]. However, these antiporter actions deplete intracellular ATP during the removal of hydrogen ions, reducing cellular viability [7,19]. Each ionophore is also selective for specific ions, and this selectivity is an index of ion-binding preference [9,22]. Although ionophores share a standard mode of action, differences in selectivity dictate the capacity of the ionophore in achieving effective rumen concentrations and their efficiency in causing bacterial changes (Table 1) [18].

Bacteria that produce ionophores are naturally insensitive to ionophores, whereas these resistance mechanisms are not well defined [23]. Insensitivity to ionophores appears to essentially result from a failure of these molecules to penetrate the bacterial cell wall, reflecting the presence of a cell membrane or extracellular polysaccharide [24,25]. Accordingly, it was proposed that ionophores preferentially inhibit Gram-positive bacteria over Gram-negative bacteria, given the penetration of these molecules into the cell membrane of these Gram-positive bacteria [3]. However, this statement is not valid for all rumen bacteria [23,25]. For instance, *Butyrivibrio fibrisolvens* is a butyric acid-producing Gram-positive bacteria insensitive to dietary ionophores [9,26]. Moreover, some Gram-negative bacteria can be initially sensitive to ionophores, and become insensitive after a period of adaptation [23,27]. In general, ionophores-sensitive bacteria are predominantly Gram-positive and produce acetic acid, butyric acid, lactic acid, and methane. In turn, ionophore-insensitive bacteria are Gram-negative bacteria that favor the production of succinate and propionate acids (Table 2) [9,28]. Despite a growing concern about Gram-positive bacteria becoming adapted and developing insensitivity to ionophores, there is limited evidence supporting this theory, which warrants further investigation [23,29].

## 3. Ionophores and Animal Production

Dietary ionophores are widely used in the beef and dairy industry as a rumen modifier and coccidiostat. Several meta-analyses are available on the effects of ionophores on beef [1,2,5,10,14,15,28] and dairy cattle performance [30]. In a meta-analyses conducted by Duffield et al. [2], monensin consistently decreased dry matter intake (DMI) by 3.1% and increased average daily gain (ADG) by 2.5% in feedlot cattle. Consequently, supplementing feedlot cattle with monensin increased feed efficiency by 1.3% [2]. These results agree with previous research conducted by Goodrich et al. [16], where cattle fed monensin-containing diets gained 1.6% more and consumed 6.4% less feed in the feedlot. Nevertheless, the improvement in feed efficiency resultant from ionophores decreased from 8.1% to 3.5% over the past 50 years, a consequence of enhanced management, nutrition, and health of feedlot cattle [2]. It is also important to note that several variables influence the difference in performance in trials using ionophores, such as days on feed, ionophore type and dose, cattle body weight, forage:concentrate ratio, type of grain fed, and type of cattle evaluated [2,14]. Golder and Lean [13] observed that cattle entering the feedlot at >275 kg and fed for a maximum of 100 days had the greatest ADG improvement in response to Lasalocid supplementation. Contrary to this, cattle with an entry weight of >275 kg that were fed lasalocid for >100 days had an intermediate increase in ADG compared with cattle with an entry weight of <275 kg, regardless of the number of days on feed [13]. Bretschneider et al. [1] observed a quadratic relationship between the dose of monensin or lasalocid and ADG in beef cattle fed forage-based diets. These authors also observed that the magnitude of the ADG response to dietary ionophores might depend on the forage quality and forage:concentrate ratio of the diet [1]. The ionophores described in this review also increased feed efficiency quadratically without affecting the DMI of grazing animals [1]. Accordingly, Limede et al. [31] reported an increase of 14.8% in ADG by adding narasin to a forage-based diet, which resulted in heavier animals at the end of 140 d supplementation period. Beck et al. [32] reported that adding monensin and lasalocid in a corn-based supplement increased ADG of grazing steers. For cattle fed grain-based diets, however, Duffield et al. [2] observed a linear effect of monensin inclusion, where greater doses improved efficiency but reduce intake and ADG response. In the review by Golder and Lean [14], lasalocid increased ADG (by an average of 40 g/d) and feed efficiency, but it did not impact the DMI of feedlot cattle. Therefore, the inclusion of ionophores in forage or grain-based diets is a beneficial management technique to optimize efficiency and performance of beef production systems. Beef producers, however, need to be aware of the differences and particularities of each ionophore to make educated decisions on the inclusion of this dietary tool in cattle diets.

## 4. Ionophores and Rumen Fermentation Function

It is well known that the inclusion of ionophores in the diet increases the feed efficiency and performance of ruminants [2,29,30] by modulating the rumen microbiome and fermentation routes and increasing energy and nitrogen efficiency metabolism [5,28]. Although ionophores available in the market have a similar mode of action in the rumen, animal performance and ruminal function may vary depending on dosage, animal, and diet [1,2,10,14]. For example, in diets containing a high concentration of readily fermentable carbohydrates (i.e., feedlot diets), ionophores generally influence feed efficiency by improving or maintaining body weight gain and reducing feed intake [1,2,5,28]. Similarly, ionophore inclusion in forage-based diets increases cattle body weight gain and feed efficiency, but with similar or increased feed intake [1,31,33,34,35]. The effects of ionophores on intake might depend on forage quality consumed by cattle, which can impact the passage rate and gut fill, and consequently intake response [1]. 

**Figure 1:**
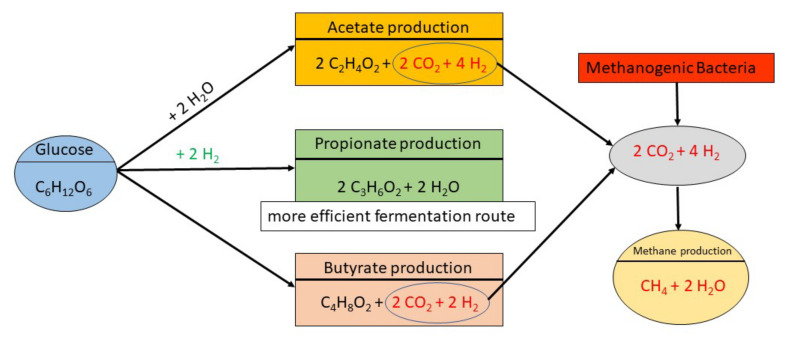
Ruminal fermentation routes and short-chain fatty acids (SCFA) and methane production. Adapted from Bergman [39] and NASEM [40].

The effects observed, at least partially, on animal performance are the response to the changes in ruminal microbiota and fermentation routes (Figure 1) promoted by the inclusion of ionophores in the diet. Approximately 75 to 85% of energy derived from the feed in the diet is converted to ruminal SCFA, and the remaining energy is lost as heat and methane [36,37]. Furthermore, 60 to 75% of ruminant’s digestible energy comes from ruminal fermentation of carbohydrates, resulting in SCFA, methane, carbon dioxide, ammonia, and microbe cells [36,38]. The predominant SCFA in the rumen are acetate, propionate, and butyrate, and their ruminal proportions are influenced by the diet [38]. In a forage-based diet, the ruminal proportions of acetate, propionate, and butyrate are generally 70:20:10, with an acetate:propionate ratio of 3:1. With a grain-based diet, the ruminal proportion of these SCFA is generally 50:40:10, with an acetate:propionate ratio of 2:1 [38].

Although all SCFA are used efficiently by the ruminant animal, propionate is the only SCFA that serves as a precursor for glucose synthesis. Propionate represents 27 to 54% of the total glucose synthesized by the liver [40], and for this reason is considered the most important SCFA fermented in the rumen [41]. Furthermore, propionate is a hydrogen sink, but acetate and butyrate are hydrogen sources, and hydrogen is the major substrate for methane formation (Figure 1) [15,42]. Methane represents an energy loss to the animal, ranging from 2% to 12% of gross energy intake [15,37]. Therefore, manipulating ruminal fermentation to produce a higher level of propionate and decreasing acetate and butyrate production is positively correlated with greater feed energy utilization and performance [1,3,7,8,28]. Additionally, an increase in propionate also mitigates methane production (Figure 1), thus improving energy efficiency obtained from the diet [1,14,15,28,38].

The inclusion of ionophores has constantly increased the ruminal concentration of propionate and reduced acetate in forage [1,31,33,34,35,43] and grain-based diets [2,5,14,28]. Accordingly, Ellis et al. [15] reported an increased proportion of ruminal propionate as the monensin dose increased in feedlot diets. Golder and Lean [14] conducted a meta-analysis to quantify the SCFA profile in beef cattle supplemented with >200 ppm of lasalocid and showed that ruminal propionate increased by 4.6% and acetate decreased by 3.2%. Polizel et al. [33] and Limede et al. [31] also reported an enhanced ruminal propionate concentration and reduced acetate and acetate:propionate ratio in beef cattle fed forage-based diets with the addition of narasin (Table 3). Moreover, monensin supplementation of steers consuming bermudagrass hay increased ruminal propionate by 10.4% and reduced ruminal acetate by 1.7% [43]. These findings support an improved energy efficiency from an increased ruminal propionate in animals fed ionophores regardless of diet. The energy density of the diet is one of the drivers for differences observed in performance and ruminal fermentation with the inclusion of ionophores in forage or grain-based diets [1,2,14,16]. Goodrich et al. [16] summarized that the optimum energy density for the inclusion of monensin in the diet is 2.9 Mcal of metabolizable energy per kg of dry matter. However, when dietary energy is lower or higher than this level, animal performance and feed efficiency responses might be reduced in response to dietary ionophores [16].

Improved energetics of rumen fermentation caused by ionophores was demonstrated by the last edition of Nutrient Requirements of Beef Cattle [40], suggesting that dietary metabolizable energy increases by 2.3% and 1.5% when monensin or lasalocid are offered to beef cattle, respectively. Accordingly, Rogers and Davis [44] reported that the total SCFA energy produced in the rumen per kilogram of dry matter consumed by steers fed a basal diet of 50% corn silage and 50% concentrate was enhanced from 0.852 Mcal/kg of dry matter for control steers to 1.137 Mcal/kg of dry matter for steers fed monensin, representing a 33% increase in digestible ruminal energy. Duffield et al. [30] reported that monensin supplementation to dairy cows effectively reduces blood concentrations of BHBA, acetoacetate, and NEFA and increases blood concentrations of glucose and urea. These findings demonstrate an improvement in the energy status of dairy cows supplemented with monensin. Therefore, ionophores successfully benefit performance by altering ruminal fermentation patterns and the energy status of ruminants.

The effects of ionophores on enhancing the rumen fermentation profile to increase propionate levels were discovered several decades ago, but drawing the principal mechanism of action has been a challenge [3]. For instance, Callaway et al. [45] reported that *Butyrivibrio fibrosolvens* is an important acetate and butyrate producer, and the capability of monensin to inhibit bacteria of the *Butyrivibrio* genus might result in improved propionate production. Accordingly, Schären et al. [4] demonstrated that administering monensin to dairy cows significantly decreased the abundance of moderate producers or non-producers of propionate. These authors also observed an increased abundance of succinate- and propionate-producing bacteria (*Prevotella* and *Ruminococcaceae*) [4]. Succinate is converted into propionate by ruminal bacteria [46], which explains, at least partially, how ionophores alter ruminal fermentation dynamics.

Ionophores inhibit methanogenesis by lowering the availability of hydrogen and formate, the primary substrates for methanogenic bacteria (Figure 1). A meta-analysis by Appuhamy et al. [47] showed that monensin supplementation reduced methane production by 2 to 15% in dairy cows and beef cattle, respectively. Schären et al. [4] reported no alteration in the abundance of methanogenic bacteria in the presence of monensin, indicating that the shift of the acetate:propionate ratio caused by ionophores reduces the substrate available to methanogenic bacteria (Figure 1), and thus decreases methane production. Another mechanism that could explain the reduction in methane production is an increase in bacteria species that compete for hydrogen [48] or a decrease in hydrogen production through the inhibition of protozoa [7].

## 5. Ionophores and Ruminal Nitrogen Metabolism

For the ruminant animal, protein and amino acid degradation in the rumen are nutritionally inefficient processes that often produce more ammonia than the bacteria can use, representing a loss of dietary nitrogen [49]. Early studies identified that the effects of ionophore supplementation on animal performance and efficiency were a reflection of the changes in ruminal microbiota and fermentation dynamics [1,2,15]. In addition, Chalupa et al. [50] suggested that part of the improvements in performance and efficiency from ionophore supplementation are resultant from decreasing ruminal proteolysis, and the accumulation of ammonia and microbial nitrogen. Several in vitro and in vivo studies observed that monensin impacts ruminal nitrogen metabolism by inhibiting deamination and proteolysis [16,49,51,52,53,54]. Therefore, a greater amount of nitrogen reaches the abomasum from the diet when ionophores are added [55,56].

Muntifering et al. [55] reported that monensin decreased the contribution of bacterial N and increased the contribution of ruminally undegraded dietary N to total abomasal N. Faulkner et al. [56] also observed that level of monensin supplementation quadratically decreased ruminal bacterial protein concentrations but increased the ruminal dietary N. According to Russel et al. [57], ionophores inhibit the production of two species of microorganisms, *Peptostreptococcus* and *Clostridium*, that have the ability to produce high concentrations of ammonia in the rumen. These species are ionophore-sensitive Gram-positive bacteria that require amino acid sources for growth; thus, dietary ionophores limit these species in the rumen, reducing deamination of dietary protein [52,57]. Accordingly, Yang and Russell [49] demonstrated that the decrease in ruminal ammonia concentration resultant from ionophores was related to a 10-fold decrease in ruminal bacteria that use amino acids and peptides as an energy source for growth. However, Golder and Lean [14] reported that administering lasalocid supplementation to beef cattle increased ruminal ammonia concentration, which contrasts the findings in other studies where the ammonia concentration decreased in monensin- or narasin-fed cattle [33,34,49,57]. Polizel et al. [33] demonstrated that administering narasin supplementation to beef cattle fed a forage-based diet for 140 d reduced the ruminal ammonia concentration by 32% compared with non-supplemented beef steers. Soares et al. [34] also reported that supplementing narasin as infrequently as every other day or daily reduced the ruminal ammonia concentration by 22% and 27%, respectively, compared with non-supplemented steers. The changes induced by dietary ionophores might result in increased ruminal peptide and amino acid concentrations, with a subsequent and consistent reduction in ruminal ammonia concentrations. The increased availability of the peptides and ammonia stimulates the growth of rumen bacteria, which can grow linearly in response to carbohydrate fermentation [58]. Collectively, the use of dietary ionophores alleviates ruminal proteolysis, reduces ammonia synthesis, and increases the influx of protein into the small intestine in cattle, which could explain, at least partially, the improvements in the performance and efficiency of beef cattle.

## 6. Ionophores’ Persistence

The effectiveness of ionophores has been documented in grain and forage-based diets [1,2,14,15,31,33,34]. However, ionophore use is limited in grazing systems due to concerns regarding depressed intake of supplements, as well as the labor required to provide supplements to cattle in extensive management [1,59,60]. The inconsistent intake of supplements by grazing cattle may also influence the effects of ionophores on rumen fermentation function and growth performance [1,34,43,60]. Meal size may also enhance the likelihood of feed additive toxicity in grazing animals, particularly if bunk space management is inadequate to prevent overconsumption [61]. Hence, the application of ionophores in grazing systems is not widespread, because most of these operations are not equipped with the resources required (bunks, carrier feed, trucks, labor, etc.) to feed cattle consistently [43].

Research has also examined the effects of ionophores, after withdrawal from the diet, on ruminal fermentation parameters, indicating a residual and long-term effect of these molecules on the proportion of SCFA, methane production, and ionophores-insensitive microbe population [17,34,43,62,63,64]. Dawson and Boling [62] observed that total ruminal SCFA in heifers supplemented with monensin only returned to basal values within 10 days after removing monensin from the diet. Rogers et al. [17] reported a 21.8% reduction in total SCFA when monensin was included in the diet of wethers for 146 days, whereas total SCFA concentration returned to basal values within 24 h of monensin withdrawal. Bell et al. [43] reported that total SCFA concentration remained 13.7% lower for 1 d in steers previously treated with monensin. By d 4 after monensin withdrawal, total SCFA concentration was similar between monensin-treated and control animals [43]. Nonetheless, these authors reported that the proportion of acetate remained lower, and that of propionate remained greater up to 7 days after monensin withdrawal compared with non-supplemented steers. A similar outcome was reported by Pasqualino et al. [64] in an ruminal environment when narasin was removed from the diet, resulting in greater proportion of propionate until 4 days after narasin withdrawal. These authors did not observe a lasting effect on the proportion of acetate, whereas the acetate:propionate ratio remained lower until day 3 after removing narasin from the diet [65]. Potchoiba et al. [63] reported that monensin maintained changes in propionate concentrations up to 3 days after removing this molecule from the diet. These results might help beef cattle producers schematizing supplementation strategies to optimize rumen fermentation parameters in grazing systems, reducing additional resources required to apply these dietary molecules. Based on this rationale, Soares et al. [34] evaluated the impacts of narasin supplementation frequency on ruminal fermentation patterns of steers fed a forage-based diet. These authors reported that decreasing the frequency of narasin supplementation from daily to every 2 days did not affect propionate, acetate, total SCFA, and acetate:propionate ratios, indicating a residual effect of this molecule in cattle receiving forage-based diets that allows infrequent supplementation to alleviate labor requirements.

It has been suggested that the use of ionophores for an extended period would also impact the persistence efficacy in ruminal fermentation response due to a possible ruminal microbial adaptation to dietary ionophores [17,66,67]. Odongo et al. [67], however, reported that monensin supplementation sustained a 7 to 9% reduction in methane production of dairy cows for 6 months. Accordingly, other previous studies demonstrated a lasting and consistent effect on ruminal fermentation parameters when monensin was fed to cattle for up to 240 days [17,65,68]. Limede et al. [31] reported increased propionate and total SCFA concentrations and reduced acetate and butyrate concentrations in steers supplemented with narasin in forage-based diets for 140 days. These authors, however, did not observe differences in ruminal fermentation parameters when salinomycin was used in forage-based diets. Other studies have shown that the reduction in ruminal methane production returned to basal levels after 30 days of supplementation [37]. Guan et al. [69] reported that monensin suppressed methane production in both high- and low-concentrate diets, whereas the duration of suppression was longer (3 weeks) when animals were fed a low-concentrate diet than when they were fed a high-concentrate diet. These results suggest that persistent effects of ionophores on ruminal fermentation patterns might be related to the diet composition, ionophore type and dose used. Nevertheless, research is warranted to validate the persistence efficacy of ionophores over a long period on rumen fermentation dynamics.

## 7. Conclusions

Ionophores are the most studied and used feed additives in beef cattle diets, with remarkably consistent evidence on altering the rumen microbiome, optimizing ruminal fermentation towards more efficient routes, reducing the rates of digestive disorders, and mitigating methane production. Differences in ruminal function likely reflect the differences in animal, diet, and type and dose of ionophore used. The effects of ionophores on ruminal fermentation dynamics appear to be consistent even with prolonged feeding periods. Moreover, the lasting impacts of the ionophore on rumen function might aid beef producers in defining nutritional strategies to improve productivity and profitability in cattle systems using this dietary technology.

## Figures and Tables

**Table 1:** Ionophores characteristics and ion-binding selectivity preference ^1^.

Ionophore	Produced by	Molecular Weight	Ion-Binding Selectivity Sequence
Monensin	*Streptomyces cinnamonensins*	671	Na^+^ > K^+^, Li^+^ > Rb^+^ > Cs^+^
Lasalocid	*Streptomyces lasaliensis*	591	Ba^++^, K^+^ >Rb^+^ > Na^+^ > Cs^+^ > Li^+^
Narasin	*Streptomyces aureofaciens*	765	Na^+^ > K^+^, Rb^+^, Cs^+^, Li^+^
Salinomycin	*Streptomyces albus*	751	Rb^+^, Na^+^ > K^+^ >> Cs^+^, Sr^+^, Ca^++^, Mg^+^

^1^ Adapted from Nagaraja [9].

**Table 2:** Sensitivity response of ruminal bacteria to ionophores.

Fermentation Products and Species	Gram Type Reaction	Sensitivity to Ionophores
Hydrogen and formic acid producers		
*Lachnospira multiparus*	Gram^+^	insensitive
*Ruminococcus albus*	Gram^+^	insensitive
*Ruminococcus flavefaciens*	Gram^+^	insensitive
Butyric acid producers		
*Butyvibrio fibrisolvens*	Gram^+^	insensitive
*Eubacterium cellulosolvens*	Gram^+^	sensitive
*Eubacterium ruminantium*	Gram^+^	sensitive
Lactic acid producers		
*Lactobacillus ruminis*	Gram^+^	sensitive
*Lactobacillus vitulinis*	Gram^+^	sensitive
*Streptococcus bovis*	Gram^+^	sensitive
Propionic and succinic acid producers		
*Anaerovibrio lipolytica*	Gram^−^	insensitive
*Fibrobacter succinogenes*	Gram^−^	insensitive
*Megasphaera elsdenii*	Gram^−^	insensitive
*Prevatella Ruminicola*	Gram^−^	insensitive
*Ruminobacter amylophilus*	Gram^−^	insensitive
*Selenomonas ruminantium*	Gram^−^	insensitive
*Succinimonas amylolytica*	Gram^−^	insensitive
*Succinivibrio dextrinosolvens*	Gram^−^	insensitive
Ammonia producers		
*Clostridium aminophilum*	Gram^+^	Sensitive
*Clostridium sticklandii*	Gram^+^	sensitive
*Peptostreptococcus anaerobius*	Gram^+^	sensitive
Methane producers		
*Methanobrevibacter ruminantium*	Gram^−^	insensitive
*Methanobacterium formicum*	Gram^−^	insensitive
*Methanosrcina barkeri*	Gram^−^	Insensitive

Adapted from Chen and Wolin [27], Russel [19], Nagaraja [9], Russel and Houlihan [23].

**Table 3:** Rumen short-chain fatty acids (SCFA) concentrations (mM/100 mM) in steers receiving forage-based diets supplemented or not (CON, *n* = 8) with narasin (NAR, *n* = 8). CON = no feed additives; NAR = inclusion of 13 ppm of narasin.

Item	Treatments			
	CON	NAR	SEM	*p–*Value
Limede et al. [31]				
Acetate	73.46	72.98	0.14	<0.01
Propionate	13.77	14.53	0.14	<0.01
Butyrate	9.05	8.60	0.10	0.01
Acetate:Proprionate	5.39	5.01	0.05	<0.01
Polizel et al. [33]				
Acetate	74.21	72.71	0.16	<0.01
Propionate	13.83	15.82	0.13	<0.01
Butyrate	8.89	8.54	0.07	<0.01
Acetate:Proprionate	5.40	4.63	0.04	<0.01
